# Supplementary material for: Transcriptomic Signatures and Molecular Pathways in Hidradenitis Suppurativa—A Narrative Review
Source: Int J Mol Sci. 2025 Aug 9;26(16):7704. doi: 10.3390/ijms26167704 (PMC12386655; doi:10.3390/ijms26167704)
Supplement: Supplementary file 1 [file ijms-26-07704-s001.zip › Supplementary Figures S1-S2.pdf]

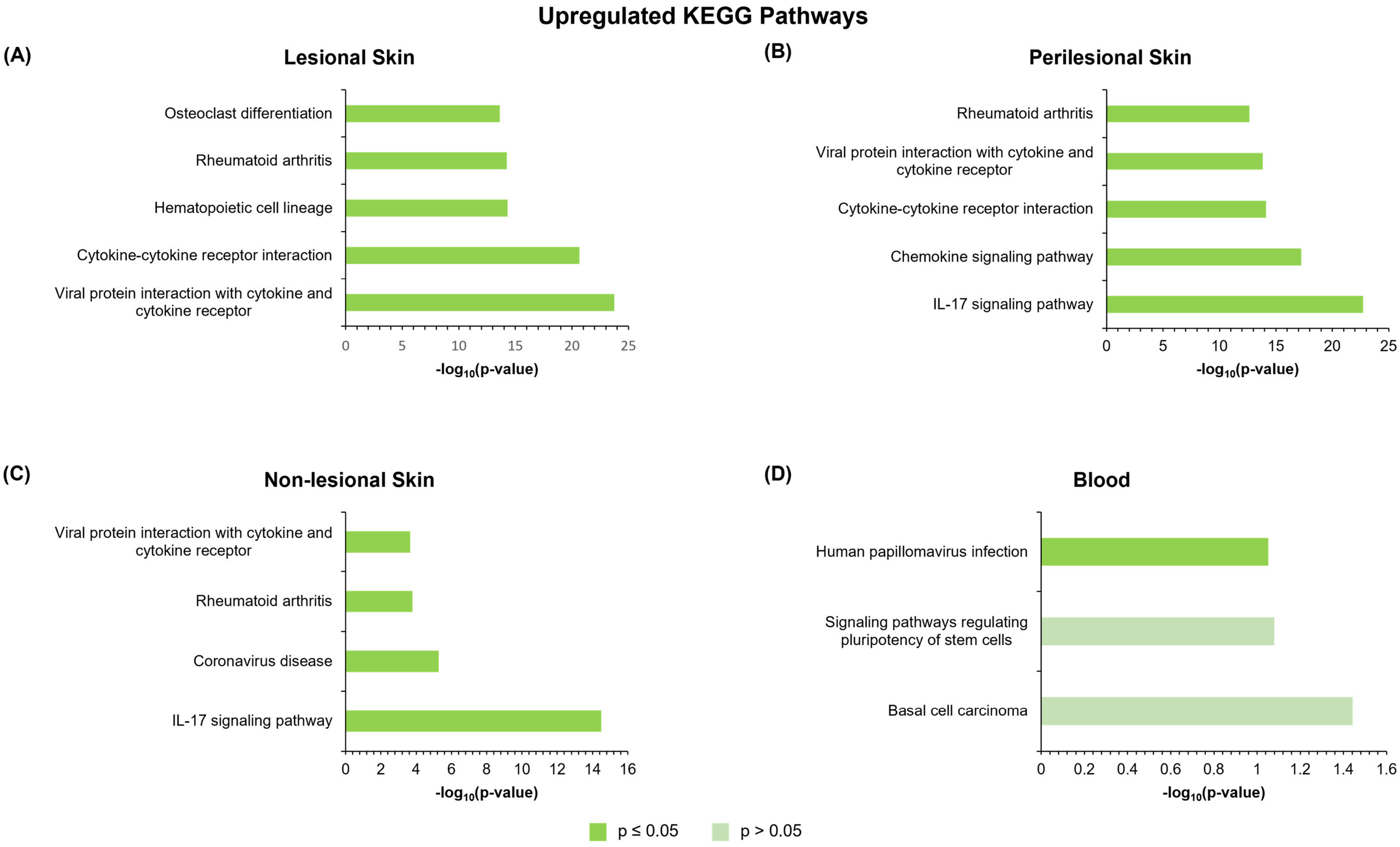

Supplementary Figure S1. Enriched KEGG pathways using Enrichr-KG in upregulated DEG lists for HS **(A)** lesional skin, **(B)** perilesional skin, **(C)** non-lesional skin, and **(D)** blood tissue when compared to healthy controls; the majority of the enriched pathways for blood tissue were statistically non-significant i.e. $p > 0.05$. The enriched pathways corroborate the results obtained using the Reactome database for over-representation analysis.

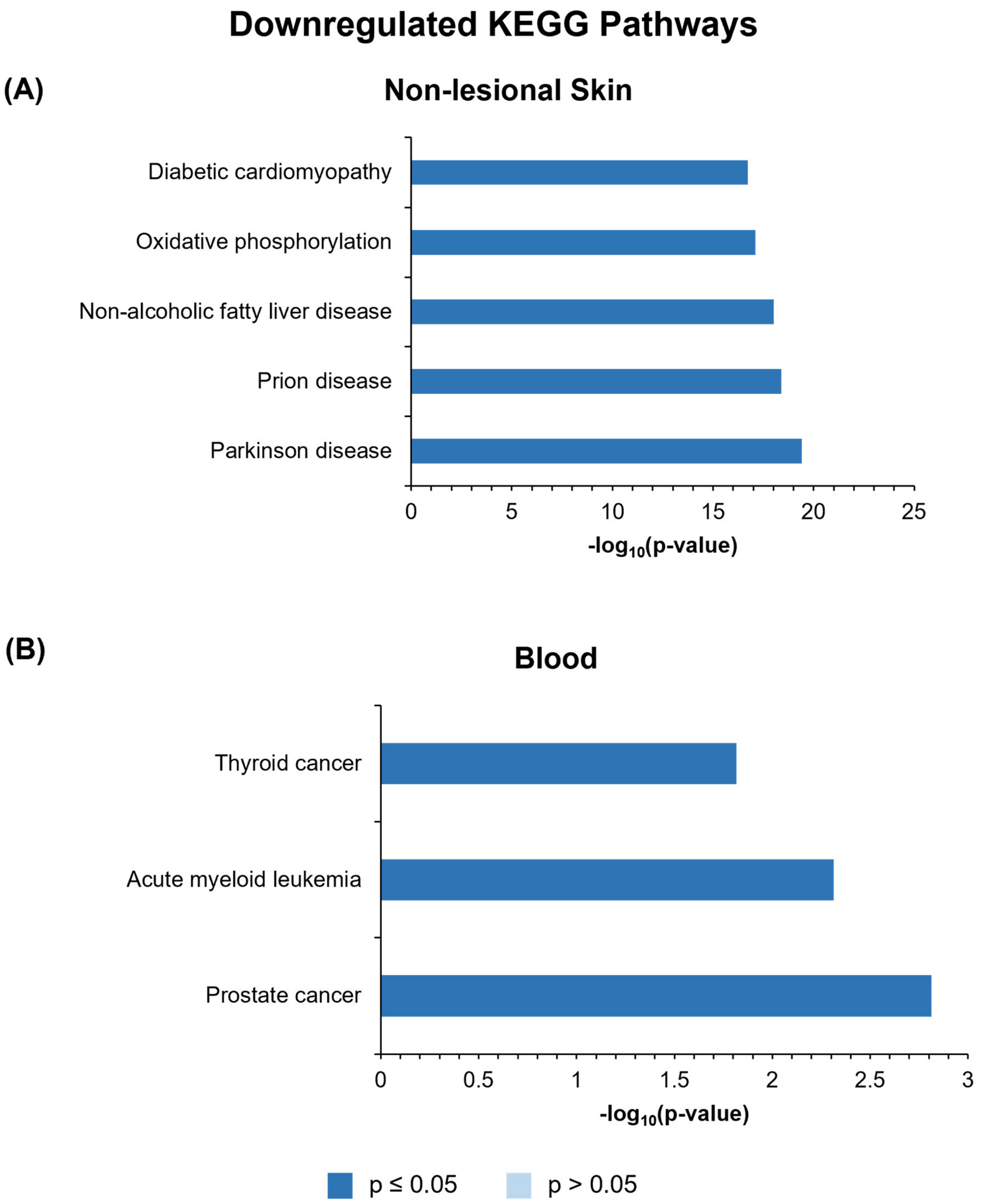

Supplementary Figure S2. Enriched KEGG pathways using Enrichr-KG in downregulated DEG lists for HS **(A)** non-lesional skin and **(B)** blood tissue when compared to healthy controls; no results were obtained for lesional skin and perilesional skin. The enriched pathways corroborate the results obtained using the Reactome database for over-representation analysis.
