# Supplementary material for: Transcriptomic Signatures and Molecular Pathways in Hidradenitis Suppurativa—A Narrative Review
Source: Int J Mol Sci. 2025 Aug 9;26(16):7704. doi: 10.3390/ijms26167704 (PMC12386655; doi:10.3390/ijms26167704)
Supplement: Supplementary file 1 [file ijms-26-07704-s001.zip › Supplementary Table 1.pdf]

Table S1. Data retrieved from the 16 selected studies, including the most prominently dysregulated signatures for each tissue type.

**Study Citation**	**Analysis Method & Threshold Value**	**Number of Study Participants (biopsy site)**		**Demographic Data**		**Number of Reported DEGs**	**Main Findings**
		**HS Patients**	**Healthy Controls**	**HS Patients**	**Healthy Controls**		
**Whole Blood** (HS patients vs healthy controls)							
Blok *et al.* 2016 [17]	Microarray *|FC| > 2, p < 0.05*	17	/	Mean age 35 ± 11 years (range: 20-53 years); mean disease duration 18 ± 11 years (range: 5-40 years); 13 female, 4 male; 13 active and 4 former smokers; mean BMI 28.3 ± 6.4 kg m^{-2} (range: 21.0-40.6 kg m^{-2}); 13 and 4 had HS Hurley stage II and III, respectively; mean Sartorius score 112.0 ± 48.6; 16 had comorbidities; all have received treatment including topical and intralesional corticosteroids, antibiotics, retinoids, biologics, as well as incision, excisions, deroofing, and intense pulse light.	/	/	No significant DEGs were identified.
Gudjonsson *et al.* 2020 [28]	RNA-Seq *|FC| > 2, p < 0.1*	20	10	Mean age 41 ± 11 years (range: 15-56 years); mean age of HS onset 25 ± 12 years (range: 8-45 years); 10 female, 10 male; 12 Caucasian, 4 Middle-Eastern, 1 North-African, 1 Surinam-Amerindian, 2 Surinam-Creole; 17 current/ex-smokers; mean BMI 32.1 ± 7.6 kg m^{-2} (range: 19.8-45.7 kg m^{-2}); 2, 14, and 4 had HS Hurley stage I, II, and III, respectively.	Mean age 72 ± 10 years (range: 55-87 years); 5 female, 4 male, 1 N/A; all Caucasian.	332	230 upregulated and 102 downregulated genes. Several DEGs increased in blood were also increased in skin, such as CCR4, TNFRSF4, and genes involved in synthesis of immunoglobulins. The top cell signatures include $CD4^+$ naïve, Th17, and Th2 cells.
**Lesional Skin** (HS patients vs healthy controls)							

Study Citation	Analysis Method & Threshold Value	Number of Study Participants (biopsy site)		Demographic Data		Number of Reported DEGs	Main Findings
		HS Patients	Healthy Controls	HS Patients	Healthy Controls		
Shanmugam *et al.* 2019 [29]	Microarray $\|FC\| > 2$, $p < 0.05$	10 (7 groin/pubic, 3 other locations)	11 (abdomen)	Mean age 35 ± 11 years; 4 female, 6 male; race: 6 African American; 8 never-smokers; mean BMI 37.1 ± 8.7 kg m^{-2}; 7 had HS Hurley stage III; mean Sartorius score 63 ± 37.3; mean VAS pain score 5.6 ± 2.3.	/	799	436 upregulated and 363 downregulated genes. The most dysregulated genes were dermcidin and IL-37, both of which were significantly downregulated. Pathway analysis identified 5 activated pathways, namely the IFN-signaling, leukocyte extravasation-signaling, Th1, Th2, and NFAT pathways.
Rumberger *et al.* 2020 [30]	Multiplex Hybridization Assay $\|FC\| > 2$, $p < 0.05$	34	16	Mean age 34 years (13-57 years); 29 female, 5 male; race: 5 black, 1 Hispanic, 7 white, 21 unknown.	Mean age 54 years (25-82 years); 7 female, 9 male; race: 10 white, 6 unknown.	129	129 upregulated genes. The most prominent were pro-inflammatory cytokines (IL-1α, TNF-α, IL-6, IL-8), as well as the IL-17, IL-10 and IFN families. Other upregulated genes included those coding for members of the JAK/STAT pathway and the tyrosine kinase-like family, antimicrobial peptides (CAMP, DEFB4A), and antibodies and members of the B-cell signaling pathways.
Gudjonsson *et al.* 2020 [28]	RNA-Seq $\|FC\| > 2$, $p < 0.1$	22	10	Mean age 41 ± 11 years (range: 15-56 years); mean age of HS onset 24 ± 12 years (range: 8 - 45 years); 11 female, 11 male; 13 Caucasian, 4 Middle-Eastern, 2 North-African, 1 Surinam-Amerindian, 2 Surinam-Creole; 19 current/ex-smokers; mean BMI 31.7 ± 7.3 kg m^{-2} (range: 19.8-45.7 kg m^{-2}); 2, 15, and 5 had HS Hurley stage I, II, and III, respectively.	Mean age 72 ± 10 years (range: 55-87 years); 5 female, 4 male, 1 N/A; all Caucasian.	4797	2584 upregulated and 2213 downregulated genes. Genes involved with B cell response were the most upregulated, such as the immunoglobulins IGLV3-27, CD19, and CD79a. Other highly upregulated genes include antimicrobial genes (DEFB4A), B cell chemoattractants (CXCL13), and neutrophil chemokines (CXCL1). The top cell signatures include B cells, T cells (Th2), and $CD4^+$ and $CD8^+$ effector memory cells. B cells and plasma are the main cell type in leukocyte infiltrates, corresponding with increased immunoglobulins and complement receptors.

Study Citation	Analysis Method & Threshold Value	Number of Study Participants (biopsy site)		Demographic Data		Number of Reported DEGs	Main Findings
		HS Patients	Healthy Controls	HS Patients	Healthy Controls		
Lowe *et al.* 2020 [31]	RNA-Seq $p < 0.05$	19	16	Age ≥ 18; moderate to severe HS with active disease involving ≥ 1 body site, but no other active skin condition; pre-TNF-α therapy.	/	4601	There is a large difference between lesional and non-lesional HS skin or healthy skin, with genes involved in B cell signaling, complement activation, T cell chemotaxis, neutrophil recruitment, macrophage activation, phagocytic pathways, IL-12 biosynthesis, and response to wounding, being significantly dysregulated. The key upregulated genes in HS inflammatory pathways were those regulated by TNF-α, IFN-γ, and IL-1β, while some immunoregulatory genes (IL-1RN, IL-10RA) and regulators of cell proliferation (α-catenin, sirtuin 1) were downregulated. The cell signature was comprised of proinflammatory M1 macrophages, activated dendritic cells, B cells, and plasma cells.
Wolk *et al.* 2021 [32]	RNA-Seq $\lvert FC \rvert > 1.5$, $p < 0.05$	30 (fresh, surgically excised skin areas)	22 (axillae or non-intertriginous sites ex. limbs)	Mean age 39 ± 11 years; 10 female, 20 male; HS Hurley stage ranging from I to III; naive to biologics.	Mean age 38 ± 11 years; 6 female, 16 male.	137	High elevation of granulocyte colony-stimulating factor (G-CSF) mRNA in lesional skin, induced by IL-17A in keratinocytes and IL-1β in fibroblasts. Comparison of samples with high vs. low G-CSF levels, showed that 124 genes were upregulated and 13 were downregulated. G-CSF was found to enhance the expression of CD16B and the membrane receptors FPR1, FPR2, FFAR2, CEACAM3, ADGRG3, LILRA1, LILRA5, and TNFRSF10C/TRAIL-R3, in neutrophils. The concentration of G-CSF in blood (although not statistically significant), correlates with the Sartorius score and number of skin sites having nodules.

Study Citation	Analysis Method & Threshold Value	Number of Study Participants (biopsy site)		Demographic Data		Number of Reported DEGs	Main Findings
		HS Patients	Healthy Controls	HS Patients	Healthy Controls		
Navrazhina *et al.* 2022 [33]	RNA-Seq $\lvert FC \rvert > 1.5$, $p < 0.05$	22 (14 axilla, 6 buttock/ groin/ inguinal, 1 neck, 1 inframammary)	10 (9 axilla, 1 buttock/ groin/ inguinal)	Mean age 37 ± 13 years; 12 female, 10 male; 14 smokers; mean BMI 31.6 ± 8.3 kg m^{-2}; 15 and 7 had HS Hurley stage II and III, respectively; mean IHS4 score 40.8 ± 65.8.	Mean age 64 ± 8 years; 8 female, 2 male; 5 smokers; mean BMI 32.1 ± 6.6 kg m^{-2}.	2871	There was an increased number of granulocyte-monocyte progenitor cells, activated myeloid dendritic cells, B cells, macrophages, and $CD4^{+}$ T cells. Significantly enriched genes were involved in B-cell signaling and complement activation, immune response, neutrophil degranulation, and extracellular matrix organization. Some of the affected immune axes include: T_H1, T_H2, T_H17, T_H22, neutrophil-related (chemotaxis, migration, extravasation) and psoriasis-related. Genes related to the IL-17 pathway were particularly activated, even in blood serum. Other highly activated markers in LCN2-high lesional samples include IFN-γ, IL-6, ICOS, THY1, CILP2, IL1B, IL36G, TNF, and CSF3.
Krajewski *et al.* 2024 [34]	RNA-Seq $\lvert FC \rvert > 1.5$, $p < 0.05$	15 (only 5 used for RNA-Seq)	6	Mean age 36 ± 11 years; 7 female, 8 male; 8 smokers; mean BMI 30.1 ± 6.31 kg m^{-2}; 2 had a family history of HS; 3, 8, and 4 had HS Hurley stage I, II, and III, respectively; mean IHS4 15.9 ± 8.9; 7 had juvenile acne during adolescence; all had not been treated for HS at least 2 months before enrolment.	/	1 (NLRP3)	A significant upregulation of NLRP3 was noted but no significant change in the expression of P2X7R.
**Lesional Skin** (Lesional vs perilesional skin from HS patients)							

Study Citation	Analysis Method & Threshold Value	Number of Study Participants (biopsy site)		Demographic Data		Number of Reported DEGs	Main Findings
		HS Patients	Healthy Controls	HS Patients	Healthy Controls		
Frings *et al*. 2022 [35]	RNA-Seq $\lvert FC \rvert > 2$, $p < 0.05$	/	/	/	/	232	183 upregulated and 49 downregulated genes were identified in lesional vs. perilesional epidermis, with enrichment of upregulated genes involved in antimicrobial response and neutrophil chemotaxis. Genes involved in T-cell regulation (such as IL4R, PDL1, and JAK3) and immune response activation (including TLR2, TLR8, TNIP3, and cGAS) were also confirmed as DEGs by RT-qPCR. Promoter sites for AP-1, STAT and Rel A were significantly enriched. Activation of STAT1 was linked to IFN-γ production and subsequent induction of immunomodulatory genes.
**Lesional Skin** (Lesional vs non-lesional skin from HS patients)							
Blok *et al.* 2016 [17]	Microarray $\lvert FC \rvert > 2$, $p < 0.05$	17	/	Mean age 35 ± 11 years (range: 20-53 years); mean disease duration 18 ± 11 years (range: 5-40 years); 13 female, 4 male; 13 active and 4 former smokers; mean BMI 28.3 ± 6.4 kg m^{-2} (range: 21.0-40.6 kg m^{-2}); 13 and 4 had HS Hurley stage II and III, respectively; mean Sartorius score 112.0 ± 48.6; 16 had comorbidities; all have received treatment including topical and intralesional corticosteroids, antibiotics, retinoids, biologics, as well as incision, excisions, deroofing, and intense pulse light.	/	745	Over 800 dysregulated genes, with a higher number of DEGs associated with increased disease duration. The modulated pathways included inflammation, immune cell signaling and communication, and atherosclerosis signaling. No changes were observed in *NCSTN*, *PSEN1* and *PSENEN* gene levels.

Study Citation	Analysis Method & Threshold Value	Number of Study Participants (biopsy site)		Demographic Data		Number of Reported DEGs	Main Findings
		HS Patients	Healthy Controls	HS Patients	Healthy Controls		
Navrazhina *et al.* 2021 [24]	Microarray *p < 0.05*	22 (14 axilla, 4 buttock, 1 groin, 1 inguinal, 1 neck, 1 inframammary)	/	Mean age 37 ± 13 years (range: 19-63 years); 12 female, 10 male; 14 smokers; mean BMI 31.6 ± 8.3 kg m^{-2} (range: 20.3-50.7 kg m^{-2}); 15 and 7 had HS Hurley stage II and III, respectively; 16 had comorbidities; prior treatments: 8 on antibiotics only, 14 on antibiotics and biologics.	/	78	The number of immune cells ($CD3^+$ T cells, $CD11c^+$ dendritic cells, and NE^+ neutrophils) in HS skin with tunnels was significantly higher, forming neutrophil extracellular traps in the tunnel lumen. Overexpression of inflammatory molecules was also noted in tunnel vs non-tunnel samples including: keratinocyte-derived factors (S100A7, S100A8, S100A9, LCN2), antimicrobial factors (DEFB4, IL-26), cytokines/chemokines enhancing neutrophil chemotaxis (CXCL1, CXCL8), neutrophil-associated factors (NCF1C, CD177), pro-inflammatory cytokines (IL-1β, GZMB, TNFRSF4, IL-6, IL-12B, IL-36α), and B-cell associated cytokines/chemokines (CD79A, TNFRSF13B, IL-20). Other upregulated molecules include: CD38, CD79A, GZMA, HLADOB, JCHAIN, LCK, TNFRS17, Keratin 6C, and Keratin 13. Conversely, anti-inflammatory molecules such as IL-37, MIF, and CCL17 were downregulated.

Study Citation	Analysis Method & Threshold Value	Number of Study Participants (biopsy site)		Demographic Data		Number of Reported DEGs	Main Findings
		HS Patients	Healthy Controls	HS Patients	Healthy Controls		
Dajnoki *et al.* 2022 [36]	RNA-Seq *p < 0.05*	10 (axilla)	/	Median age 28 years; 5 female, 5 male; moderate to severe HS; biological therapy naïve.	/	23	Keratinocyte-related mediators/sensors (except for LCN2 and CAMP), cell surface markers and TH1/Th17-related mediators and transcription factors (such as TBX21) were significantly upregulated. RORC was downregulated. These differences were not significant when comparing the tissues using RT-qPCR and IHC. However, the number of T cells, DCs and macrophages was significantly higher in the dermis, as was the overexpression of the Th1/Th17-related mediators CD83, IFNG, IL-17A, IL-10, IL-23A and TGFB1, and IL-12$^+$, IFN-γ^+, TNF-α^+, IL-17A$^+$, IL-10$^+$, IL-23$^+$, TGF-β$^+$ and CCL20$^+$ cells.
Flora *et al.* 2023 [37]	RNA-Seq $\lvert FC \rvert \geq 1.5$, $p \leq 0.05$	20	/	Mean age 35 ± 10 years (range: 20-52 years); 11 female, 9 male; 13 and 7 had HS Hurley stage II and III, respectively.	/	6424	Fibroblast-related genes were enriched in lesional skin, as indicated by the upregulation of THY1, LUM, FN1, and COL1A1. Gene signatures for SFRP2$^+$ fibroblasts were upregulated, whereas those for SFRP1$^+$ were downregulated; there was no significant difference in CXCL12$^+$ gene signatures. IHC showed that the increase in SFRP2$^+$ and CXCL12$^+$ cells is associated with more severe HS and the presence of epithelialized tunnels. In addition, epithelial mesenchymal transition was observed in lesional skin, particularly in the presence of tunnels.

Study Citation	Analysis Method & Threshold Value	Number of Study Participants (biopsy site)		Demographic Data		Number of Reported DEGs	Main Findings
		HS Patients	Healthy Controls	HS Patients	Healthy Controls		
Freudenberg *et al.* 2023 [21]	RNA-Seq $\lvert FC \rvert \geq 3$, $p \leq 0.1$	20 (4 axilla, 9 inguinal, 2 buttock, 1 inframammary, 1 labia majora, 1 pubic, 2 other)	/	Mean age 36 ± 10 years; mean age at diagnosis 32 ± 11 years; mean age of HS onset 23 ± 11 years; 17 female, 3 male; 13 white, 7 non-white; 5 smokers, 6 former smokers, 9 non-smokers; mean BMI 36.4 ± 11.6 kg m^{-2}; 8 had a family history of HS; mean IHS4 score 10.2 ± 12.7; treatment at sampling time: 7 no treatment, 8 used antibiotics, 2 on diuretics, 1 used 5-alpha reductase inhibitor, 1 on oral contraceptives, 1 on retinoids.	/	1430	1430 DEGs were identified. The top upregulated genes included matrix metalloproteinases (MMP1, MMP2, MMP3, MMP9, MMP12), cytokines/chemokines (CXCL5, CXCL13, IL-12, IL-23, IL-17, IL-36, IL-6, IL-1B), antimicrobial peptides (SERPINB4, S100A12, S100A15, S100A7, S100A8, DEFB4A), members of the complement pathway (C5AR1, CR1, FCN1). The downregulated genes included those associated with sweat gland function (prolactin induced protein, secretoglobins, aquaporin 5/AQP5), antimicrobial peptides (DCD), cytokines (IL-1A, IL-18), proteolytic enzymes (ELANE). Joint reanalysis of HS transcriptomes identified 118 consistently dysregulated protein coding genes mostly involved in immune pathways (inflammation, granulocyte activation, neutrophil degranulation, B cell, T cell and complement activation, IL-10 signaling, and ECM organization.

Study Citation	Analysis Method & Threshold Value	Number of Study Participants (biopsy site)		Demographic Data		Number of Reported DEGs	Main Findings
		HS Patients	Healthy Controls	HS Patients	Healthy Controls		
Ben Abdallah *et al.* 2023 [22]	RNA-Seq $\|FC\| \geq 2$, $p \leq 0.01$	15 (inguinal or axilla)	/	No topical or systemic treatment for 2-4 weeks prior to biopsy collection.	/	3654	2636 upregulated and 1018 downregulated genes were identified. The most upregulated were immunoglobulin and immune-related genes, matrix metallopeptidases, antimicrobial peptides, and genes related to skin structure. Inflammatory processes were the most enriched, such as B cell, T cell and complement activation, neutrophil chemotaxis, and antimicrobial response. The cell-type signature had increased numbers of $CD3^+$ and $CD8^+$ T cells, B cells, $CD11c^+$ macrophages/dendritic cells, myeloperoxidase$^+$ neutrophils. Compared to psoriasis and atopic dermatitis, HS exhibited overexpression of $T_H1/2/17$ gene signatures (with no preference towards one T_H signature) and a more widespread activation of the immune system.
**Perilesional Skin** (HS patients vs healthy controls)							
Navrazhina *et al.* 2022 [33]	RNA-Seq $\|FC\| > 1.5$, $p < 0.05$	22 (14 axilla, 6 buttock/ groin/ inguinal, 1 neck, 1 inframammary)	10 (9 axilla, 1 buttock/ groin/ inguinal)	Mean age 37 ± 13 years; 12 female, 10 male; 14 smokers; mean BMI 31.6 ± 8.3 kg m^{-2}; 15 and 7 had HS Hurley stage II and III, respectively; mean IHS4 score 40.8 ± 65.8.	Mean age 64 ± 8 years; 8 female, 2 male; 5 smokers; mean BMI 32.1 ± 6.6 kg m^{-2}.	2987	No DEGs were identified between perilesional and lesional samples. Perilesional samples were enriched with genes similar to those found in lesional skin and shared similar cell signatures and immune axes.
**Non-lesional Skin** (HS patients vs healthy controls)							

Study Citation	Analysis Method & Threshold Value	Number of Study Participants (biopsy site)		Demographic Data		Number of Reported DEGs	Main Findings
		HS Patients	Healthy Controls	HS Patients	Healthy Controls		
Lowe *et al*. 2020 [31]	RNA-Seq *p* < 0.05	13	16	Age ≥ 18; moderate to severe HS with active disease involving ≥ 1 body site, but no other active skin condition; pre-TNF-α therapy.	/	1495	Approximately 2300 identified DEGs, the main differences related to genes involved in homeostasis and components of the myeloid compartment. IL-1R2 and CD163 were significantly reduced, indicating the reduction in activated M2 macrophages and conventional dendritic cells. Other downregulated genes were antimicrobial peptides, CD14, TLR4, and TLR7. Notably upregulated genes were TNF-α, CSF1, IFNLR1. TNF-α production by $CD8^+$ T cells and $CD4^+$ Tcons was similar in non-lesional and lesional skin, indicating that immune responses are elicited before signs of inflammation are noted.
Navrazhina *et al*. 2022 [33]	RNA-Seq $\lvert FC \rvert > 1.5$, $p < 0.05$	22 (14 axilla, 6 buttock/ groin/ inguinal, 1 neck, 1 inframammary)	10 (9 axilla, 1 buttock/ groin/ inguinal)	Mean age 37 ± 13 years; 12 female, 10 male; 14 smokers; mean BMI 31.6 ± 8.3 kg m^{-2}; 15 and 7 had HS Hurley stage II and III, respectively; mean IHS4 score 40.8 ± 65.8.	Mean age 64 ± 8 years; 8 female, 2 male; 5 smokers; mean BMI 32.1 ± 6.6 kg m^{-2}.	310	Enriched genes were mainly involved in early epidermal development/reprogramming. The IL-17 signature is also activated in non-lesional skin.

**Study Citation**	**Analysis Method & Threshold Value**	**Number of Study Participants (biopsy site)**		**Demographic Data**		**Number of Reported DEGs**	**Main Findings**
		**HS Patients**	**Healthy Controls**	**HS Patients**	**Healthy Controls**		
Dajnoki *et al*. 2022 [36]	RNA-Seq<br>$p < 0.05$	10 (axilla)	8 (axilla)	Median age 28 years; 5 female, 5 male; moderate to severe HS; biological therapy naïve.	Median age 52 years; 8 female.	9	There was no significant difference in immune cells surface markers (CD4, CD11c, CD83, CD163) and Th1/Th17-related mediators and transcription factors, except for the downregulation of TGFB1. Some keratinocyte-related mediators, namely the antimicrobial peptides S100A7, S100A8, S100A9, were overexpressed, whereas TLR4 was downregulated. The latter have also identified IL-1β in the dermis, TNF-α in the apical epidermis, and IL-23 in the basal keratinocyte layers of the epidermis, as significantly upregulated. S100A8, LCN2, and hBD-2 were upregulated in the interfollicular epidermis but not significantly.
Krajewski *et al*. 2024 [34]	RNA-Seq<br>$\lvert FC \rvert > 1.5$, $p < 0.05$	15 (only 5 used for RNA-Seq)	6	Mean age 36 ± 11 years; 7 female, 8 male; 8 smokers; mean BMI 30.1 ± 6.31 kg m^{-2}; 2 had a family history of HS; 3, 8, and 4 had HS Hurley stage I, II, and III, respectively; mean IHS4 15.9 ± 8.9; 7 had juvenile acne during adolescence; all had not been treated for HS at least 2 months before enrolment.	/	1 (NLRP3)	A significant upregulation of NLRP3 was noted using RNA-Seq, but the increase in expression was not corroborated by RT-PCR. There was no significant change in the expression of P2X7R.
**Single Cell** (Derived from lesional HS skin vs healthy control skin)							

Study Citation	Analysis Method & Threshold Value	Number of Study Participants (biopsy site)		Demographic Data		Number of Reported DEGs	Main Findings
		HS Patients	Healthy Controls	HS Patients	Healthy Controls		
Gudjonsson *et al.* 2020 [28]	scRNA-Seq *$p \leq 0.1$*	5	/	Mean age 44 ± 6 years (range: 36-53 years); mean age of HS onset 21 ± 13 years (range: 15-45 years); 2 female, 3 male; 4 Caucasian, 1 North-African; 5 current/ex-smokers; mean BMI 30.2 ± 6.6 kg m^{-2} (range: 21.9-39.0 kg m^{-2}); 5 HS Hurley stage III.	/	/	10 major cell types were identified: keratinocytes, melanocytes, fibroblasts, smooth muscle cells, endothelial cells, B cells, plasma cells, T cells, myeloid cells, and mast cells. Keratinocytes were the largest cell population, with 13 identified sub-clusters. Of these, only a proportion responded to inflammatory stimuli in HS, with IFN-γ being the predominant response. Leukocyte infiltration, with a predominant plasma cell and B cell signature, was higher in HS lesions compared to normal skin.
Lowe *et al.* 2020 [31]	scRNA-Seq *$p < 0.05$*	12	14	Age ≥ 18; moderate to severe HS with active disease involving ≥ 1 body site, but no other active skin condition; 10 pre-TNF-α therapy.	/	/	The most enriched cell populations were neutrophils in HS skin as opposed to type 2 conventional dendritic cells (cDC2) and $CD163^{+}$ macrophages in healthy skin. The levels of these cells varied during the different stages of disease; for example, cDC2 and Langerhans cells decreased before end-stage disease, while $CD163^{+}$ macrophages decreased during the acute/chronic inflammatory phase but increased again at end-stage. IL-1β, CXCL8, PTGS2, and NFKBIA were also significantly increased in cDC2s in lesional skin. In addition, B cells were also found to differ with disease stage - these increased as skin lesions involved, resulting in more differentiated memory B cells, plasma cells, and plasmablasts at end-stage.

Study Citation	Analysis Method & Threshold Value	Number of Study Participants (biopsy site)		Demographic Data		Number of Reported DEGs	Main Findings
		HS Patients	Healthy Controls	HS Patients	Healthy Controls		
Mariot-toni *et al.* 2021 [25]	scRNA-Seq *p ≤ 0.05*	3 (axilla)	1	Mean age 43 ± 21 years (range: 27-66 years); 3 female; 3 African-American; 3 had HS Hurley stage III.	Age 48; 1 male; African-American.	6619	A gene signature enriched for IFN signaling, macrophage function and antimicrobial activity with downregulation of sweat gland-associated genes. Overall, innate immune processes related to phagocytosis and Fc receptor-mediated signaling were enriched. Thus, the most common cells in HS were immune cells such as monocytes/macrophages, dendritic cells, Langerhans cells, B/T cells. Focusing on macrophages, the pro-inflammatory M1-like macrophages were favored, with genes involved in phagocytosis, respiratory burst and ADCC activation being upregulated (MHC class II, STAT1, GBP5, FCGR1A/B, FPR1/2, SOD, ITGB2, GZMK). Conversely genes associated with the anti-inflammatory M2 macrophages were downregulated (CD163, MRC1). These genes are stimulated by both type I and type II interferons. Antiviral and antimicrobial proteins were also highly upregulated in dermal monocytes/macrophages, but were even more pronounced in plasmacytoid dendritic cell (pDCs).

Study Citation	Analysis Method & Threshold Value	Number of Study Participants (biopsy site)		Demographic Data		Number of Reported DEGs	Main Findings
		HS Patients	Healthy Controls	HS Patients	Healthy Controls		
Jin *et al.* 2023 [38]	scRNA-Seq $\lvert FC \rvert > 2$, $p < 0.05$	8	8 (breast or abdomen)	5 female, 5 male; 2 Caucasian, 8 black.	7 female, 1 male; 3 Caucasian, 5 black.	1826 (keratinocytes)	Comparison of the transcriptomes in HS and the eIF4A signature showed that 516 upregulated and 218 downregulated genes were shared between the two: the top upregulated genes were related to tumorigenesis (ACTRT3, CNN3, PTPRN2), inflammation and innate immunity (IL10RA, CBARP, PTAFR, TLR7, BTK), keratinocyte differentiation and keratinization (CRABP2, CCR2, FLI1), and skin fibrosis (DOCK10); enriched downregulated genes were associated with Wnt signaling (WNT2B, WLS), epidermal homeostasis and cell adhesion (KLK1, WDR72, LEFN2), and modulation of inflammation (TPH1, CXCL14). Through pathway enrichment analysis, it was noted that signals associated with the eIF4 complex regulated inflammatory, immune and oncogenic signaling processes which enhance the risk of tumorigenesis. This is further supported by the increased presence of actively proliferating epithelial cells as well as the overexpression of the oncogenes *CCND1* and *MYC* in the epidermal layer.
